# Surgery

**Published:** 1894-10

**Authors:** 


### SURGERY.

EDITED BY FLOYD W. McRAE, M.D.

### POINTS TO BE REMEMBERED IN RECTAL SURGERY.

1. Remember, To operate upon all cases of fistula where there is sufficient vitality or nutrition to heal the wound, always dividing the fibrous membrane at the bottom of tract and packing wound to the bottom, for the purpose of healing by granulation.

2. Remember, To always open abscess early to prevent fistula in ano.

3. Remember, If you operate on fistula, in a tubercular patient, give him the benefit of a doubt.

4. Remember, That you should never fail to examine your patient thoroughly, for small arms leading out from the main track, and examine for an associate stricture which may be the cause of the fistulous tract.

5. Remember, That you are never to cut the sphincter but once in any operation, and be careful to warn your patient of the danger of incontinence.

6. Remember, That you are to confine your patient in bed, not trusting to the care of a nurse (exclusively). Tuberculous cases should be an exception to the rule—giving them moderate exercise and fresh air.

7. Remember, That physiological rest is the first principle in the cure of all diseases.

8. Remember, That varicosed veins are not hemorrhoids until they have passed through the stage of inflammation and plastic exudation.

9. Remember, That hemorrhoids that don't protrude don't demand an operation, as a rule.

10. Remember, That uncomplicated hemorrhoids have no pain.

11. Remember, That there is a differential diagnosis to be made between hemorrhoids, prolapse of the bowel, and polypus.

12. Remember, That the ligature is simple in application, free of danger, and certain in its results.

13. Remember, That you are to transfix the base of the large pile, and not the small one, cutting off two-thirds, and tying tightly to prevent hemorrhage.

14. Remember, That there are two forms of external piles: one you can cut off, the other you can ligate.

15. Remember, That you are always to remove all complications at the time of removing the hemorrhoids.

16. Remember, That you are to remove all external tagis during the operation for internal hemorrhoids.

17. Remember, The capillary or strawberry pile, for danger may come to the lot of your patient.

18. Remember, The Mathews tampon, which is simple in construction and easy in application.

19. Remember, Hysteria to be a disease when applied in rectal surgery, having for its causes a change in quantity or quality of blood and a change in the solids consequent thereon.

20. Remember, The reflexes in diseases of the rectum, for you will find a pahological cause somewhere along the telegraphic line of the lumbar or sacral plexus.

21. Remember, That an operation on disease in the contiguous organ will relieve the protean symptoms in the rectum.

22. Remember, In connection with the reflexes the small fistulous tract beneath the mucous membrane, the sensitive nerve, the denuded epithelium, and nothing but patience and perseverance by the use of the probe will reveal the organic lesion in question.

23. Remember, The irritable ulcer or fissure, located on the sphincter muscle, caused by a denudation of the epithelium, does not heal for the want of physiological rest.

24. Remember, Divulsion cures it.

25. Remember, Ulcers in the rectum are oftener caused by constipation, and a locked-up liver action, than any other known cause.

26. Remember, That ulcers in the rectum are not caused, as a rule, by dysentery.

27. Remember, That when you give them physiological rest and remove the cause, or pull out the nail, as Professor Carpenter has so thoroughly impressed upon your mind, they will heal.

28. Remember, The sigmoid flexure as a receptacle, and liable to ulcerations, which you may cure by medicines of a disinfecting and cleaning nature, to be used through a Wales bougie.

29. Remember, The classification of stricture: acquired, the result of inflammation; congenital, which is not a stricture proper, but an atresia, the result of an arrest of development.

30. Remember, That there are simple tubular or valvular strictures of a benign nature, that are amenable to treatment by gradual dilatation, to be used indefinitely, or we might say as long as the patient has an existence.

31. Remember, One half of all the strictures in the rectum are syphilitic.

32. Remember, That in all malignant strictures of the rectum nothing short of colotomy, inguinal preferred, will spare your patient's life for a short time , and no operation, only when suggested by the patient.

33. Remember, Syphilitic stricture in its incipiency should be treated constitutionally.

34. Remember, Tuberculosed strictures, where the lungs are badly involved, are to be let alone.

35. Remember, That all strictures, no matter where located, are the result of inflammation, with plastic effusion or infiltration.

36. Remember, That prolapsus ani is a disease confined more especially to childhood, and where it occurs in the adult it is the lingering result of childhood misfortune.

37. Remember, That you may mistake prolapsus for hemorrhoids, and only a clear understanding of an anatomy of the parts will lead you to a differential diagnosis.

38. Remember, You are to select Mathews's method for relief in the adult; palliative astringents, with strapping for children.

39. Remember, Pruritus ani is the result of nervo-reflex actions and filthy habits.

40. Remember, That it is the most formidable of all diseases, as far as cure and treatment are concerned.

41. Remember, That you should relieve the cause, and by the use of campho-phenique and temperance, or total abstinence from the use of alcoholic drink and tobacco, you may cure the disease.

42. Remember, The rules that you are to be governed by, quantity and quality of blood, physiological rest, thorough asepsis and antisepsis.—DR. OATMAN, in *St. Louis Medical Era.*

---

### THE SURGERY OF THE GALL-BLADDER AND BILE-DUCTS, WITH BRIEF NOTES OF SEVENTY-EIGHT CASES.

Robson (*British Medical Journal*, April 28, 1894) speaks of the most prominent symptoms and complications of cases of cholelithiasis which have come under his observation :

1. Spasms or biliary colic without jaundice, the attacks being repeated at longer or shorter intervals.
2. Collapse due to the intensity of the pain.
3. Spasms followed by evanescent jaundice.
4. Pain followed by persistent jaundice and enlargement of the liver.
5. Hydrops of the gall-bladder without jaundice.
6. Ileus due to atony of the bowel.
7. Acute intestinal obstruction due to paralysis of the gut from local peritonitis, volvulus of the small intestine, or impaction of a large gall-stone in some part of the intestine.

In the majority of these cases, where medical treatment has failed, surgical procedures hold out very good hope of success in nearly every complication, if the patient be not too much exhausted.

Cases complicated with malignant disease are very unfavorable for operation. First, because the subjects of cancer are, as a rule, cachectic and worn down by disease before the surgeon is called in; and, secondly, because such patients are prone to hemorrhage at the time of the operation or subsequently.

The author claims that there is considerable risk in operating on patients that are markedly jaundiced, on account of hemorrhage, but more especially as the jaundice is frequently associated with malignant disease. In order to avert the danger of hemorrhage in jaundiced patients, the author has found that the administration of chloride of calcium for a few days before the operation makes the blood more plastic and lessens the tendency to bleeding at the time of the operation and subsequently.

In jaundiced cases he prefers ligating all bleeding points rather than trust pressure forceps.

In all of these cases operated upon by the author, in which there was malignant disease with jaundice, the gall-bladder formed a perceptible tumor; whereas, when the jaundice was dependent upon gall-stones, no marked tumor was present. A valuable diagnostic point, the author claims, is tenderness on pressure over some point between the eighth or ninth costal cartilage and the umbilicus.

The so-called diagnostic operation of sounding for gall-stones and aspiration of a distended gall-bladder the author believes to be futile and dangerous, and is much better replaced by a small exploratory incision, when treatment can at the same time be carried out, if required.

The indications for operating in these cases the author gives as follows:

1. Frequently recurring biliary colic, without jaundice, with or without enlargement of the gall-bladder.
2. Persistent jaundice ushered in by pain.
3. Empyema of the gall-bladder.
4. Peritonitis starting in the region of the liver.
5. Purulent collections about the gall-bladder.

If the bladder and ducts can be cleared without much difficulty the opening in the gall-bladder can be sutured to the aponeurosis and drained.

If the ducts cannot be cleared, one of the following procedures may be carried out:

(*a*) Cholelithotrity, the stone being crushed between the finger and thumb or by padded forceps.

(*b*) Choledodectomy, opening the duct, removing the stone, and suturing the duct afterwards. A drainage-tube should always be inserted in the right kidney pouch in these cases.

(*c*) Cholecystenterostomy. This operation my be easily performed if the gall-bladder be dilated. The author prefers the decalcified bone bobbin, as only two sutures must be applied.

(*d*) The daily injection of fluids after an interval of some days, through the cholecystotomy opening, which will either soften or dissolve the concretion. For this, hot water, or a solution of taurochlorate soda, may be used, or as the author prefers injection of olive oil or a five per cent. solution of oleic acid.

(*e*) Cholecystectomy may be required as a secondary operation in cases of stricture of the cystic duct, the common duct being free.

With but very few exceptions a vertical incision along the upper part of the right semilunar line gives ample room.—G. C. DAVIS, M.D., in *University Medical Magazine.*

---

### ON THE MORTALITY AFTER OPERATIONS FOR STRANGULATED HERNIA; THE TREATMENT OF GANGRENOUS INTESTINE, AND THE RADICAL CURE OF HERNIA."

In this paper Mr. Croft gives the results of the operations for hernia performed by him from May, 1866, to October, 1892. During this period he operated for strangulated hernia ninety-four times, forty-four of the operations having been performed since the introduction of antiseptic methods. The mortality of the fifty cases operated upon in preantiseptic days was over fifty per cent., as compared with a mortality of twenty-nine and five-tenths per cent. in more recent years. The author agrees with Mr. Bowlby that "most of the deaths after operation for strangulated hernia are due to exhaustion, resulting from compulsory starvation of several days' duration, as well as from continuous retching, vomiting, and pain." He has analyzed the causes of death in the thirteen cases in which the operation was performed antiseptically, and finds that in nine death was due to exhaustion from protracted sufferings and old age, and that in none was peritonitis the cause of the fatal result. Of the patients operated upon in preantiseptic days, sixteen died of "peritonitis or exhaustion from protracted suffering and feeble old age," and five from erysipelas or pyemia.

In ten cases, all of which died, it was thought necessary to make an artificial anus. In only two does the author consider that resection and suture of the gut could have been entertained for a moment. Mr. Kendal Franks, who has collected two hundred and twenty cases of resection of the gangrenous intestine, considers that the rule should be to resect the affected portion of gut, reserving the formation of an artificial anus for special cases. Omentum was present in the sac in thirty-five of the ninety-four herniæ, and a greater or less portion of it was removed in twenty-seven.

The author, in a large proportion of his cases, completed the operation of herniotomy by performing radical cure, and thinks that whenever possible this should be the routine treatment.—JOHN CROFT, in *American Journal of the Medical Sciences*, April, 1894. *Medical Chronicle.*

---

### THE TREATMENT OF INOPERABLE MALIGNANT TUMORS WITH THE TOXINES OF ERYSIPELAS AND THE BACILLUS PRODIGIOSUS.

Dr. Wm. B. Coley, attending surgeon to the New York Cancer Hospital, has an interesting paper on this subject in the July issue of the *Am. Jour. Med. Sci.* Dr. Coley has been engaged in this form of investigation for a number of years and the results, as he cites a number of cases in the present paper—which is really a continuation of a previous one in 1892—and his statements are so clearly put that we are tempted to quote rather freely. After mentioning the more particular forms of sarcoma yielding to the toxines the writer says :

"All of the above cases have been kept under constant observation. When we consider that they were all hopeless cases from an operative standpoint, or from any hitherto known method of treatment ; that in every case the diagnosis was not only established by eminent surgeons, but also confirmed by the microscopic examination of competent pathologists, then only are we in a position to properly estimate the importance of this subject.

In regard to cases of carcinoma we must, at present, speak with more reservation. In none of the eight cases which I have treated did the tumor entirely disappear, yet the very marked improvement that occurred in the most unpromising cases has strengthened me in the belief that the future treatment of inoperable carcinoma as well as sarcoma lies in this direction. The method of treatment is yet in its infancy, and there is every reason to believe that it can be greatly improved by careful experiment and research."

The following conclusions are then offered :—

"1. The curative action of erysipelas upon malignant tumors is an established fact.

2. This action is much more powerful in sarcoma than carcinoma.

3. This action is chiefly due to the toxines of the erysipelas streptococcus, which may be isolated and used with safety.

4. This action is greatly increased by the addition of the toxines of bacillus prodigiosus.

5. The toxines to be of value should come from virulent cultures and should be freshly prepared.

6. The results obtained from the use of toxines without danger are so nearly quite equal to those obtained from an attack of erysipelas, that inoculation should rarely be resorted to."—*Chicago Clinical Review.*

---

MANAGEMENT OF THE INTESTINE AFTER ABDOMINAL SECTION.

Drs. Skene Keith and George E. Keith (New York *Journal of Gynecology and Obstetrics*) says:

When the general condition of the patient is fairly good and the abdomen is not distending, and when there is not much colic, let things take their natural course. This advice holds for the great majority of cases.

When the abdomen is distending without a previous state of peristaltic action shown by much colic, and when there is a tendency to vomiting, give the mixture of magnesia until the bowels move.

Treat distention without colic or vomiting by the quinine injections.

Quiet excessive peristaltic action by small doses of morphine given hypodermatically.

For distention following excessive peristalsis, give a small dose of morphine followed in a few hours by castor oil or magnesia.

When there is septicemia, wash out the abdomen as soon as the condition is evident and treat as above.

Do not get into the habit of calling septicemia shock, exhaustion, or any such term. Be content to believe that some mistake has been made in the antiseptic precautions and that additional care must be taken in the future.—*Times and Register.*

### NARCOSIS STATISTICS: CHLOROFORM, ETHER, ETC.

Professor Gurlt lately made a report on this subject to the German Surgical Society, 69 members of the Society sending paticulars. The total number of narcoses reported was 52,475—*viz.*, 33,083 with chloroform, 11,669 with ether, 3,896 with ether and chloroform, 750 with mixture of alcohol, chloroform, and ether, 2,986 with ethyl bromide, and 91 with nitrous oxide. If the last are subtracted, the gas was used in tooth extractions, there are 52,384 cases of anethesia, with the very considerable number of 21 deaths—1 in 2,494. In the reports given six other deaths are noted as being due to the anesthetic, but as they occurred two to thirty hours *after* the operation, they can hardly be considered as resulting from the action of the anesthetic. The results of four years of observation are: Chloroform narcosis, 166,812 cases, with 63 deaths—1 in 2,647; ether, 26,320, with 2 deaths—1 in 13,160; mixed chloroform and ether, 8,014, with 1 death; alcohol, chloroform, ether, 4,190, with 1 death; ethyl bromide, 7,541, with 2 deaths; and dental, 597 cases, with 3 deaths—1 in 199. The mortality in chloroform, moreover, in this past year has been very high, 17 deaths being reported in the 33,083 cases—1 in 1,946. Chloroform has been used in somewhat fewer cases; the use of the ether has increased. In two of the fatal cases Pictet chloroform was used. With respect to method of administration, the slow-drop method was most largely used. In nine cases paralysis is reported as occurring after the narcosis. Although ether is the least dangerous agent used, it is reported as causing a greatly increased flow of saliva and mucus, and often followed by bronchitis or bronchopneumonia. The full reports are given, and will be of great interest to those concerned in the administration of anesthetics.—*Medical Chronicle.*

---

### THE RADICAL CURE OF HERNIA.

Dr. F. C. Larimore (*Journal of the American Association*) says: The operation for the radical cure of hernia is a safe operation. Mortality, 0.86 per cent.

The operation results in a permanent cure, relapses occurring in but 7.5 per cent. of the cases.

The operation should be made with the strictest aseptic technique.

Make a free incision over and with the long axis of the tumor, from without inward.

Open the sac and examine the contents deliberately and carefully, generally resecting the omentum.

Dissect up the sac, ligate its neck high up with a chain suture, and resect it below the suture.

Restore the obliquity of the inguinal canal by two rows of deep, continuous double sutures.

Close the integument with a buried suture.

For all sutures use kangaroo tendon, and do not draw so tight as to constrict the tissues.

Final dressing: Iodoform collodion.

Keep patient in bed from three to seven days.

Do not put on a truss.—*Times and Register.*

---

### CAN ONE ACQUIRE GONORRHEA FROM A PERSON WHO HAS IT NOT?

Morel-Lavallée (*Annales des Maladies des Organes Génito-Urinaires*, December, 1893, p. 905) has an article with the above heading. He gives a case in which a husband, wife, and lover were involved, and submits the following conclusions:

Viewed clinically: (1) The morbific agent of gonorrhea may rest latent for months, or even years (in one of his observations six years). (2) The blennorrhagic virus may, in the woman, remain absolutely latent for three or four weeks, and still preserve its virulence. (3) The occurrence of the menses seemed to facilitate the outbreak of the affection.

Viewed socially: It is impossible to permit marriage in a man that has the slightest drop of discharge until it has been proved by the tests of Neisser (nitrate of silver), or bichloride of mercury, or beer (Janet), to be aseptic.

Viewed bacteriologically: (1) The gonoccocus can be deposited in the genital tract and not develop, the patient escaping the disease entirely. (2) It may rest in the genital tract and still retain its virulence and capability of transmitting the disease. (3) The gonococcus may remain in the female urethra, at least for several days, without giving rise to any appreciable clinical signs. (4) Menstruation may light up a gonorrhea which would otherwise have remained latent. The author ends by answering the question with which he heads his article by quoting Ricord, to the effect that "the most beautiful girl in the world can only give that which she has." —*University Medical Magazine.*

---

### WHEN MAY SYPHILITICS MARRY?

In a late paper Dr. W. S. Gottheil, of New York, attempts to answer this question, according to the following conclusions:

1. Syphilitics may not marry:

(*a*) During the entire primary stage, from first appearance of the chancre to the entire disappearance of the induration and the inguinal adenopathy. This stage will last at least six months. Secondary symptoms will almost always appear during its continuance. and it will run into the next one. If they do not, a further delay of twelve months is necessary to await them.

(*b*) During the entire secondary stage, the period of the general eruptions, mutiple mucous patches, adenopathies, alopecias, etc. This stage lasts at least twelve months, and is rarely prolonged over two years. But it is not the time so much as the generalized character of the disease manifestations, showing persistent infection. and prolonged contagiousness, that marks its limits.

2. Syphilitics may marry:

(*a*) When the disease is in its tertiary stage. This stage lasts from the disappearance of the general symptoms to the end of the patient's life. He may or may not have symptoms of the disease or its sequelæ, but it is no longer contagious, and he cannot communicate it to his partner.

(*b*) When a period of one year has elapsed since the appearance of the last symptom of the disease.

